# Bobby Sox homology regulates odontoblast differentiation of human dental pulp stem cells/progenitors

**DOI:** 10.1186/1478-811X-12-35

**Published:** 2014-05-30

**Authors:** Young-Ae Choi, Mi-Youn Seol, Hong-In Shin, Eui Kyun Park

**Affiliations:** 1 Department of Oral Pathology and Regenerative Medicine, School of Dentistry, Kyungpook National University, 2177 Dalgubeol-daero, Jung-gu, Daegu 700-412, Korea

**Keywords:** BBX, Transcription factor, DPSC

## Abstract

**Background:**

Transcription factors have been implicated in regulating the differentiation of odontoblasts from dental pulp stem cells/progenitors (DPSCs/progenitors), but their regulatory network is not completely understood.

**Result:**

New transcription factors that control the odontoblast differentiation of human DPSCs/progenitors were analyzed using a microarray. The result revealed bobby sox homolog (BBX) to be expressed most strongly during odontoblast differentiation. Validation using RT-PCR also revealed the strong expression of BBX during the odontoblast differentiation of DPSCs/progenitors. BBX expression was also detected in adult molar odontoblasts and other tissues, including the heart, kidney, testis, and bone marrow. To understand the role of BBX in odontoblast differentiation, *BBX* variant 1 and 2 cDNA were cloned and overexpressed in DPSCs/progenitors. The results showed that the overexpression of *BBX* cDNA in DPSCs/progenitors induced substantial mineralization and expression of the odontoblast marker genes, such as *ALP*, *OPN*, *BSP*, *DMP1*, and *DSPP*. The knockdown of *BBX* using shRNA, however, did not affect mineralization, but the expression of *ALP* and *DSPP* was decreased substantially. Meanwhile overexpression or knockdown of BBX did not modulate proliferation of DPSCs/progenitors.

**Conclusion:**

Our results suggest that BBX plays an important role during the odontoblast differentiation of human DPSCs/progenitors.

## Background

Human DPSCs/progenitors have been identified in dental pulp tissue [1], and harbor the characteristics of plastic adherence and the expression of stem cell markers, such as CD29, CD90, CD44 and CD146 [1]. DPSCs/progenitors have the capacity to differentiate into a range of cell types *in vitro*, including odontoblasts [2], osteoblasts [1,3,4], chondroblasts [1,3,4], adipocytes [4], neuronal cells [5,6], and hepatocyte-like cells [7]. DPSCs can form dentine-like tissue both *in vitro* and *in vivo*[8,9]. DPSCs possess a higher population doubling time, and neural and epithelial stem cell properties than bone marrow mesenchymal stem cells [10,11].

Interactive transcription factors have been implicated in regulating cellular responses of stem cells. DPSCs express some of the transcription factors expressed in embryonic stem cells, including Oct-4, Sox-2 and Nanog [12-14]. These transcription factors may regulate the proliferation, self-renewal and differentiation of DPSCs. Oct-4A translocates from the nucleus to the cytoplasm during differentiation of DPSCs [15]. This nucleus-to-cytoplasm shuttling of Oct-4A is associated with phosphorylation [15]. In the case of Nanog, it is localized in the nucleus of undifferentiated DPSCs, whereas the cytoplasmic and nuclear localization are reduced in the differentiated cells [15]. Thus, nucleus-to-cytoplasm shuttling of some transcription factors is a key mechanism regulating commitment or differentiation of DPSCs.

Other transcription factors also play key roles in the odontoblast differentiation of DPSCs. For example, during tooth development, the increased expression of type 1 collagen and Dspp is correlated with the persistent expression of Twist1 and the down-regulation of Runx2 [16,17]. In human DPSCs, the overexpression of TWIST1 stimulates the expression of the late-mineralization markers, such as OCN, DMP1 and OPN, which are expressed in both odontoblasts and osteoblast differentiation. TWIST1 enhances the expression of dentin sialophosphoprotein (DSPP), a dentin-specific marker, suggesting that TWIST1 regulates the odontoblast differentiation of DPSCs [18]. In addition, KLF4 regulates the odontoblast differentiation of dental pulp cells. KLF4 overexpression up-regulates the activity of alkaline phosphatase (ALP) and the expression of mineralization-related genes, including ALP, dentin matrix protein 1 (DMP1), and dentin sialoprotein (DSP). These results suggest that KLF4 can promote the odontoblast differentiation of DPCs [19].

Therefore, a growing number of transcription factors that act positively or negatively on odontoblast differentiation of DPSCs/progenitors have been identified. However, the genetic and regulatory networks of transcription factors involved in odontoblast differentiation are not completely understood. Here, we have utilized microarray and subsequent functional analysis to identify a new transcription factor, BBX, which regulates odontoblast differentiation of human DPSCs/progenitors.

## Results

### Microarray analysis of gene expression in DPSCs/progenitors undergoing odontoblast differentiation

To examine the transcriptional regulation involved in the odontoblast differentiation of human DPSCs/progenitors, the gene expression profile of DPSCs/progenitors undergoing odontoblast differentiation was analyzed by microarray analysis. The DPSCs/progenitors were isolated, as described in the Materials and methods, and found to be positive for CD29, CD44, CD105 and CD90 [20]. The cells were cultured in differentiation media for 7 days, and the total RNA was isolated at days 1 and 7. The microarray was conducted with the Affymetrix HGU133 Plus2 GeneChip Array. The transcription-related genes were first selected based on the gene ontology, and the genes showing more than 2.5 fold higher expression in differentiating DPSCs/progenitors than the undifferentiated control at day 7 were selected. Table 1 lists the selected genes. *BBX* was the most strongly expressed gene during the odontoblast differentiation of human DPSCs/progenitors.

**Table 1:** List of the genes expressed strongly during the odontoblast differentiation of DPSCs/progenitors (>2.5 fold at day 7)

**Gene symbol**	**Gene title**	**RefSeq**	**DPSC**	
		**Transcript ID**	**1 d**	**7 d**
BBX	Bobby sox homolog (Drosophila)	NM_020235	3.03	6.50
SATB1	SATB homeobox 1	NM_002971	0.93	4.92
ZBTB38	Zinc finger and BTB domain containing 38	NM_001080412 /// XM_001133510 /// XM_001133669	1.62	4.59
ZBTB20	Zinc finger and BTB domain containing 20	NM_015642	1.52	4.59
ZFP90	Zinc finger protein 90 homolog (mouse)	NM_133458	1.15	4.59
PBX1	Pre-B-cell leukemia homeobox 1	NM_002585	1.52	4.29
SSBP2	Single-stranded DNA binding protein 2	NM_012446	1.23	3.73
CREB1	cAMP responsive element binding protein 1	NM_004379 /// NM_134442	2.14	3.03
PPARG	Peroxisome proliferator-activated receptor g	NM_005037 /// NM_015869 /// NM_138711 /// NM_138712	1.32	3.03
ZNF12	Zinc finger protein 12	NM_006956 /// NM_016265	1.62	3.03
IRF2	Interferon regulatory factor 2	NM_002199	1.74	2.83

### Validation of microarray data

**Figure 1:**
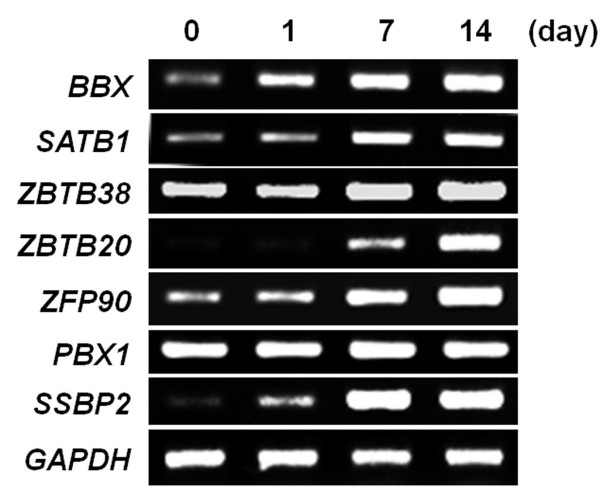
**Validation of the microarray data.** The expression levels of the top 7 genes in DPSCs/progenitors undergoing odontoblast differentiation. The total RNA was isolated at the indicated time, and the expression of the genes was analyzed by RT-PCR. All experiments were performed in triplicate.

To validate the microarray data, several genes were selected and their expression during odontogenic differentiation was analyzed by RT-PCR. The results showed that almost all selected genes showed increased expression during the odontoblast differentiation of DPSCs/progenitors (Figure 1). These results demonstrate that the microarray data are reliable. Of the genes tested, the expression of *BBX* was increased from day 1 after the odontogenic medium treatment. BBX belongs to the high mobility group (HMG) family and is related to the SOX family of proteins, which play a key role in the proliferation and lineage commitment of mesenchymal stem cells [21,22]. Therefore, this study examined whether BBX is involved in proliferation and odontoblast differentiation of DPSCs/progenitors.

### Isoform and tissue distribution of BBX

**Figure 2:**
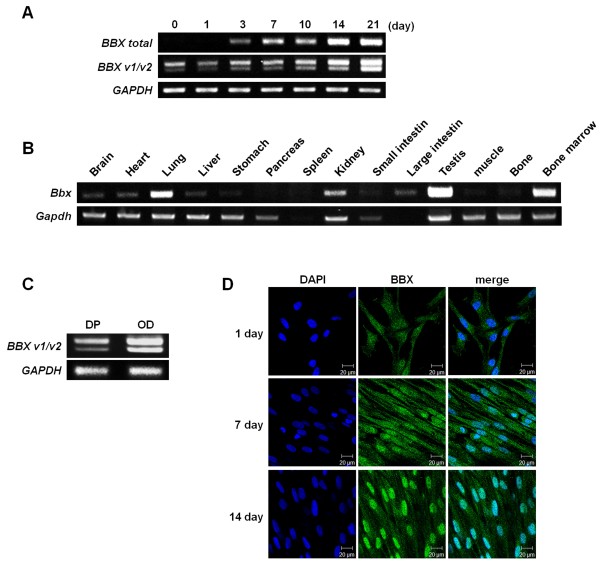
**Isoforms of BBX and its expression in various tissues.** PCR primers were designed to detect both the total and isoforms of BBX. **(A)** The expression of the BBX isoforms during the odontoblast differentiation of human DPSCs/progenitors showed that variant 1 is dominantly expressed. **(B)** Tissue expression of Bbx was analyzed by RT-PCR. The total RNA was extracted from the tissues of adult mice, and the expression of *Bbx* was analyzed by RT-PCR. **(C)***BBX* expression was also analyzed in human molar odontoblasts (DP: dental pulp, OD: odontoblast). **(D)** BBX was immunostained with anti-BBX antibody at day 1, 7 and 14. scale bar: 20 μm. All experiments were performed in triplicate.

Human BBX has three variant forms. *BBX* variant 1 is 90 base pairs longer than variant 2. *BBX* variant 3 is 1009 bp shorter than variant 1. The expression of the *BBX* variants during odontoblast differentiation was confirmed using primers that can detect three variants (Additional file 1: Table S1). As shown in Figure 2A, *BBX* variant 1 was expressed as the major form in the DPSCs/progenitors, but variant 2 was also increased by odontogenic medium stimulation (Figure 2A). However, BBX variant 3 was not detected during odontoblast differentiation of DPSCs/progenitors (data not shown). The expression pattern of *Bbx* in various tissues was also examined. The results showed that *Bbx* was expressed in the heart, kidney, testis, and bone marrow in an adult mouse (Figure 2B). These results suggest that Bbx might be involved in the development or functional regulation of those tissues. In addition, *BBX* expression was examined in the odontoblast layer and dental pulp tissue of human molars (Figure 2C). *BBX* was strongly expressed in the odontoblasts compared to dental pulp tissue. *BBX variant 1* was dominantly expressed in human odontoblasts. Immunofluorescence staining of the cultures was performed at days 1, 7, and 14 (Figure 2D). The results showed that BBX was localized to both the nucleus and cytoplasm until day 7. However, BBX was noticeably translocated into the nucleus at day 14. These results suggest that BBX plays an important role in odontoblast differentiation of human DPSCs/progenitors.

### Enhanced odontoblast differentiation of DPSCs/progenitors by BBX overexpression

**Figure 3:**
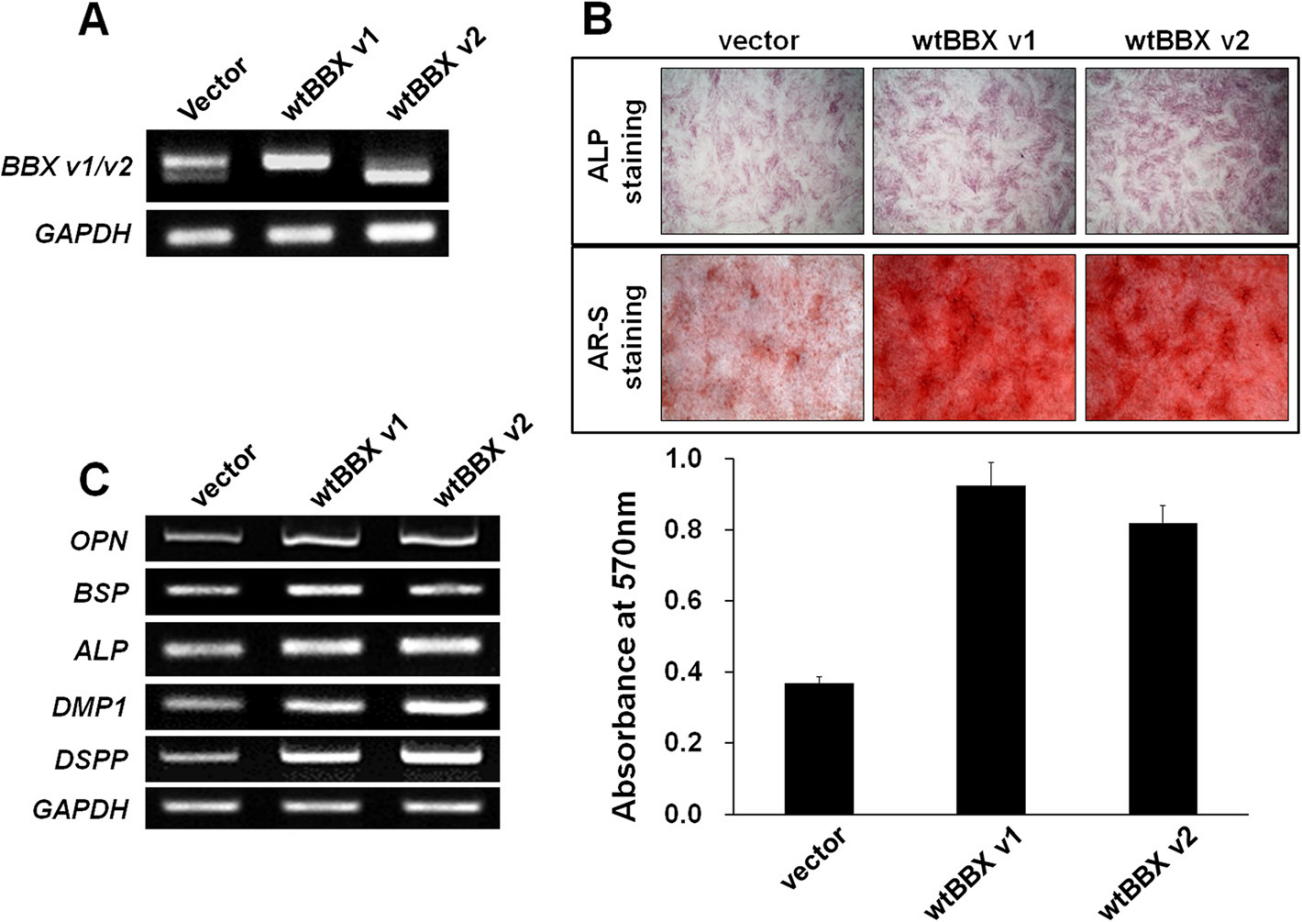
**Odontoblast differentiation of DPSCs/progenitors by BBX overexpression. (A)** BBX variant 1 and 2 were overexpressed using electroporation, and their expression was confirmed by RT-PCR. **(B)** ALP activity (on day 10) was increased by BBX overexpression (upper panel). Mineralization was assessed by alizarin red S staining and BBX overexpression induced an increase in mineralization (on day 25) compared to the controls (middle panel). The alizarin red S stain was extracted and the OD was measured (lower graph). **(C)** The expression of several odontoblast differentiation markers was analyzed by RT-PCR, and they were increased in the BBX-overexpressing cells than in the empty vector-expressing cells on day 14. All experiments were performed in triplicate.

DPSCs/progenitors undergoing odontoblast differentiation and human odontoblasts expressed BBX. Therefore, we examined whether BBX regulates odontoblast differentiation and mineralization of DPSCs/progenitors. The full length cDNA for *BBX* variant 1 and 2 were cloned, and tagged with the FLAG sequence at the C-terminal. *BBX* variant 1 and 2 cDNAs were electroporated to DPSCs/progenitors using a microporator. The overexpression of each variant was confirmed by RT-PCR at 1 day following electroporation (Figure 3A). Although very weak, expression of exogenous BBX was detected up to 14 days after electroporation (Additional file 2: Figure S1). ALP staining was performed at day 10 because *ALP* expression is an early event for odontoblast differentiation. The ALP staining level was increased significantly in the DPSCs/progenitors overexpressing wild type *BBX* variant 1 and 2 (Figure 3B upper panel). In addition, the effect of *BBX* overexpression on mineral deposition was also examined. At day 25, the cells overexpressing *BBX* cDNAs were strongly stained with alizarin red S (Figure 3B middle panel). The Alizarin red S stain was extracted from the cultures and quantified using a spectrophotometer at 570 nm (Figure 3B lower graph). The results showed that the overexpression of *BBX* variant 1 and 2 stimulated mineral deposition by 2.5-and 2.1-fold, respectively, compared to the control. The expression level of the marker genes related to odontoblast differentiation and mineralization were analyzed to further confirm odontoblast differentiation at day 14 (Figure 3C). Consistent with ALP staining, the overexpression of *BBX* cDNAs induced ALP gene expression at day 14. The expression of BSP was only partially increased, whereas *OPN* expression was induced. Importantly, the expression of *DMP1* and *DSPP*, which are odontoblast differentiation markers, were increased significantly by *BBX* overexpression. These results suggest that BBX may participate in the odontoblast differentiation of DPSCs/progenitors.

### Suppression of odontoblast differentiation of DPSCs/progenitors by BBX knockdown

**Figure 4:**
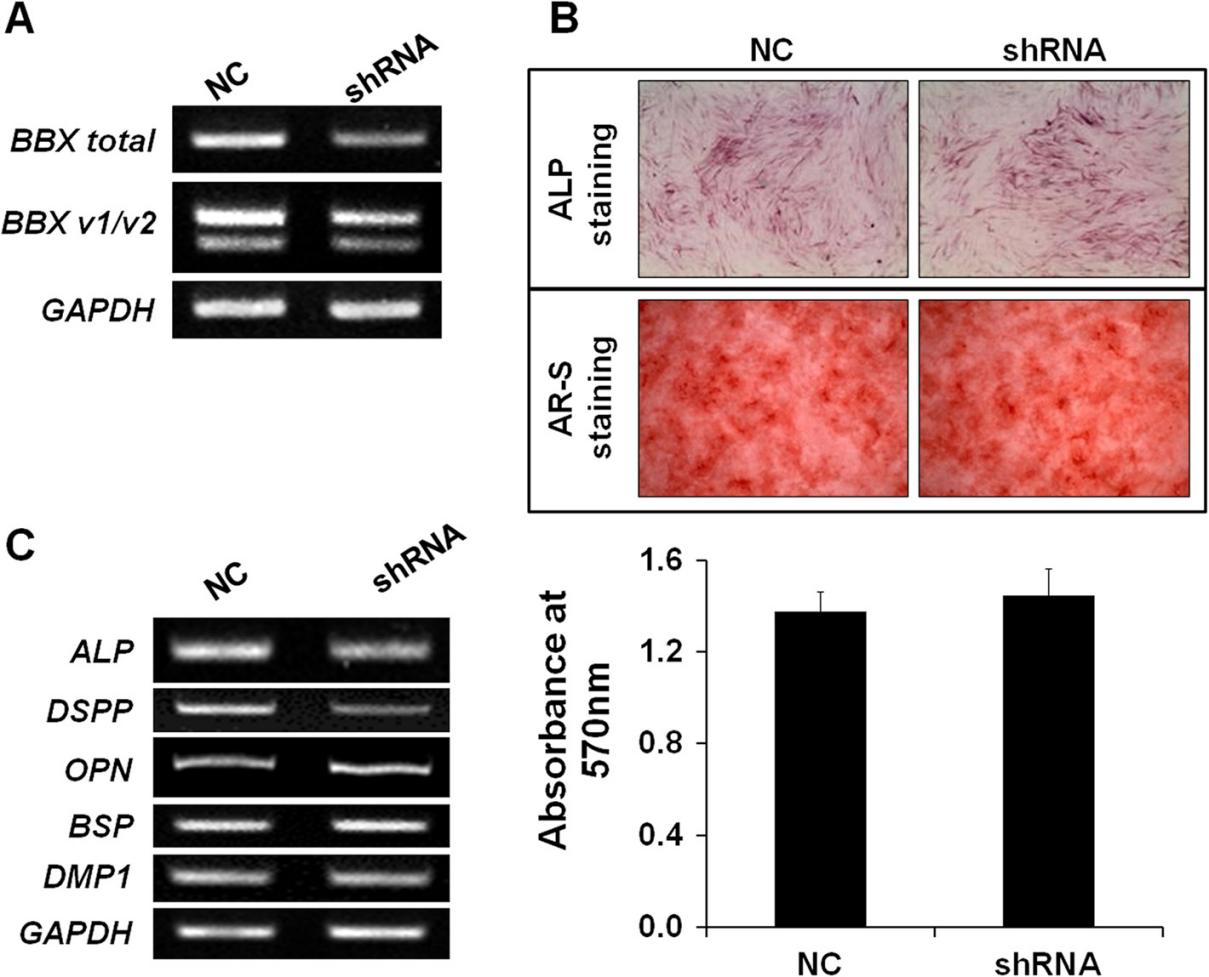
**Odontoblast differentiation of DPSCs/progenitors by BBX knockdown. (A)** The BBX shRNA was electroporated, and BBX expression was confirmed by RT-PCR. **(B)** ALP staining and mineralization was not decreased substantially by BBX shRNA (upper and middle panel). The alizarin red S stain was extracted and the OD was measured (lower graph). **(C)** The expression of several odontoblast differentiation markers was regulated by BBX knockdown. Note that ALP and DSPP was decreased significantly by BBX shRNA. All experiments were performed in triplicate.

To examine the effect of the knockdown of BBX on odontoblast differentiation, BBX shRNA was electroporated into the DPSCs/progenitors, and odontoblast differentiation was induced. BBX shRNA decreased the expression of endogenous BBX substantially (Figure 4A). The DPSCs/progenitors electroporated with BBX shRNA were stimulated with the odontogenic medium, and ALP and Alizarin red S staining were performed. Interestingly, BBX knockdown did not result in a decrease in ALP activity and mineral deposition (Figure 4B upper and middle panels). The quantification of Alizarin red S staining did not show significant changes between the control and BBX shRNA expressing DPSCs/progenitors (Figure 4B lower graph). An analysis of odontoblast marker expression showed that the expression of *OPN*, *BSP,* and *DMP1* were not inhibited significantly by BBX shRNA (Figure 4C). On the other hand, the expression of *DSPP* was inhibited dramatically by BBX shRNA (Figure 4C). This suggests strongly that BBX knockdown suppresses the odontoblast differentiation of DPSCs/progenitors, but it only partially affects the expression of other mineralization-related genes.

### Growth of DPSCs/progenitors by BBX

**Figure 5:**
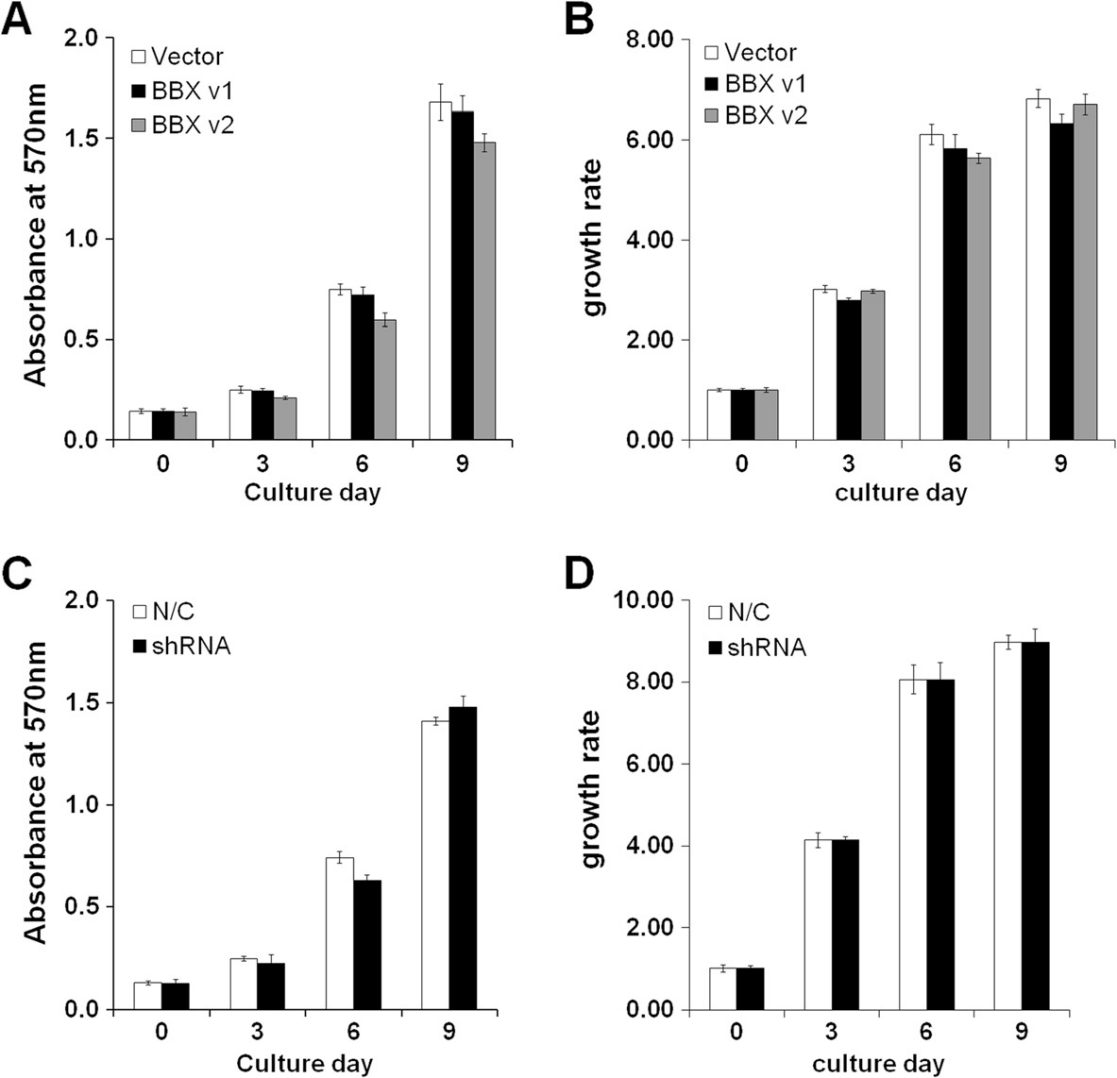
**Proliferation of DPSCs/progenitors by the overexpression and knockdown of BBX. (A and B)** BBX variants 1 and 2 cDNAs were electroporated into the DPSCs/progenitors, and cell proliferation was measured using MTT and cell counting at the indicated culture days. **(C and D)** BBX shRNA was electroporated, and proliferation of DPSCs/progenitors was measured using MTT and cell counting at the indicated culture days. All experiments were performed in triplicate.

A previous study reported that BBX is involved in the G1/S transition in yeast [23]. Therefore, we examined whether BBX overexpression or knockdown can modulate the proliferation of DPSCs/progenitors. DPSCs/progenitors were electroporated with vector alone or *BBX* variant 1 or 2, and proliferation was analyzed using a MTT assay for up to 9 days of culture (Figure 5A). The results showed that the overexpression of BBX variant 2 reduced cell growth only partially from day 3, the changes in the OD ranged only ~ 10%. However, to confirm the effect of BBX on cell proliferation, the number of cells were counted at the indicated time. As shown Figure 5B, cell numbers were not significantly changed by BBX overexpression (Figure 5B). Moreover, BBX shRNA did not affect cell proliferation as assessed by MTT and cell counting (Figure 5C and D). These results suggest that BBX may not affect cell growth of human DPSCs/progenitors.

## Discussion

The molecular mechanisms of odontoblast differentiation were studied by examining the *in vitro* odontoblast differentiation of DPSCs and *in vivo* mouse tooth developmental model. During tooth development, significant numbers of genes have been shown to regulate odontoblast differentiation, and they function either synergistically or counteractively to induce the formation of dentin and teeth [24]. In the *in vitro* DPSCs/progenitors system, several transcription factors have been shown to regulate the odontoblast differentiation. This study identified BBX as a new transcriptional regulator for the odontoblast differentiation of human DPSCs/progenitors.

Using microarray and validation, several transcription factors were found to be expressed selectively during odontoblast differentiation of human DPSCs/progenitors (Table 1 and Figure 1). For example, Special AT-rich sequence-binding protein (SATB) was highly expressed during odontoblast differentiation. SATB overexpression induces osteoblastic differentiation and mineralization matrix deposition in MC3T3-E1 cells [25]. The expression of other transcription factors was also increased. These include *ZBTB36, ZBTB20, ZFP90,* and *SSBP2*. Since *BBX* was the most highly expressed gene in DPSCs/progenitors undergoing odontoblast differentiation of DPSCs/progenitors, we further examined whether BBX is involved in proliferation and odontoblast differentiation of DPSCs/progenitors.

The most differentially expressed gene in DPSCs/progenitors was BBX (Table 1 and Figure 1). BBX is a HMG box containing transcription factor known as HMG box-containing protein 2 (HBP2). HMG-box domains have been identified as being members in the diverse group of DNA-binding proteins including transcription factors [26]. High mobility group proteins contain the HMG-box domains, and are involved in the regulation of DNA-dependent processes, namely transcription, replication, and DNA repair [27]. On the other hand, the role of BBX in relation to tooth development or stem cell differentiation has not been reported. Recently, BBX was reported to be a target of nuclear factor I X (Nfix), and Nfix represses the expression of BBX during cortical development [28]. Mice lacking Nfix exhibit a delay in the differentiation of the neural progenitor and an expansion of the progenitor population within the hippocampus and neocortex, suggesting that Bbx regulates neuronal progenitor cell renewal and cell differentiation [29]. Mammalian Bbx, also called as HBP2, has been shown to promote the G1/S transition in the fission of yeast [23]. In DPSCs/progenitors, however, the overexpression or knockdown of BBX only moderately affected proliferation (Figure 5), suggesting that BBX only partially contributes to the proliferation of mesenchymal stem cells. Therefore, BBX does not seem to directly regulate cell cycle transition in DPSCs/progenitors. These results do not exclude a role of BBX in stem cell growth/survival in other tissues, and further studies will be needed to better understand the molecular mechanisms regulating cell cycle progression in neuronal progenitors.

The expression of BBX is increased during the odontoblast differentiation of DPSCs/progenitors (Figures 1 and 2D) and detected in odontoblasts of human molar (Figure 2C). During odontoblast differentiation, BBX localization to the nucleus appears to be increased (Figure 2D). These results suggest that BBX expression and translocation to the nucleus is increased during odontoblast differentiation of DPSCs/progenitors. Importantly, the overexpression of BBX induced the odontoblast differentiation of DPSCs/progenitors (Figure 3). In addition, BBX knockdown suppressed the expression of DSPP substantially (Figure 4), suggesting that BBX plays an essential role in the odontoblast differentiation of DPSCs/progenitors. It is also interesting to note that BBX regulates the expression of DSPP, although considering the time gap, the regulation appears to be indirect. In addition, overexpression of BBX stimulates the mineralization process whereas its downregulation does not. Although we do not have direct evidence, this might be due to functional redundancy of BBX in mineralization process. On the other hand, BBX was also expressed in BMSCs undergoing osteoblast differentiation (Additional file 3: Figure S2). These results are consistent with a recent report, showing that Bbx is involved in the regulation of bone mass [30]. Bbx null mice were produced and characterized by the European Conditional Mouse Mutagenesis Program [30,31]. The Bbx mice have dental asymmetry and low bone mineral density, suggesting that Bbx might regulate tooth and bone development. Therefore, these results, showing the regulatory role of BBX in the odontoblast differentiation of DPSCs/progenitors, are consistent with the dental phenotype of Bbx knockout mice. Bbx null mice also showed a lower nose to tail length. The retardation of growth might be due to the impaired growth of bone or poor feeding because of dental problems. Therefore further investigation is required to determine the cause of this phenotype.

Several transcription factors have been shown to regulate the odontoblast differentiation of human DPSCs/progenitors. For example, the lentiviral transduction of TWIST1 in DPSCs induces odontoblast differentiation and mineralization of DPSCs. In addition, the knockdown of TWIST1 reduces the *Dspp* promoter activity significantly [18]. KLF4 overexpression also up-regulates the activity of ALP and the expression of mineralization-related genes, including ALP, DMP-1, and dentin sialoprotein (DSP) [19]. These results suggest that TWIST1 and KLF4 can regulate the odontoblast differentiation of DPSCs. Therefore, future studies should examine whether BBX is functionally related to TWIST and KLF4. Because RUNX2 has antagonistic action with TWIST1 during the odontoblast differentiation of DPSCs, it is also interesting to determine whether there is a functional interaction of BBX with RUNX2 in the odontoblast differentiation of DPSCs.

## Conclusion

In summary, the genome-wide expression of DPSCs/progenitors undergoing odontoblast differentiation were analyzed, and several genes that are expressed specifically during odontoblast differentiation were identified. The role of BBX was analyzed further, through overexpression and knockdown of BBX. The results show that BBX plays a key role in the regulation of odontoblast differentiation of human DPSCs/progenitors. Future studies aimed at the mechanism of action of BBX in odontoblast differentiation, will provide a further understanding of the role of BBX in odontoblast differentiation and dentin formation.

## Materials and methods

### Cell cultures

Sound third molars extracted from patients for orthodontic treatments at the Kyungpook National University Hospital were collected after informed consent was obtained and signature of a consent forms. Isolation of DPSC cells and all experimental procedures were approved by the Institutional Research Board of Kyungpook National University Hospital (KNUH BIO_081007). Human DPSCs/progenitors were isolated and cultured as described previously [20]. Briefly, the tooth surfaces were cleaned and cut around the cementum–enamel junction using a dental engine to reveal the pulp chamber. The pulp tissues were separated gently from the crown and root, and digested in a solution containing 3 mg/mL type I collagenase and 4 mg/mL dispase (Gibco Ltd., Uxbridge, UK) for 45 minutes at 37°C. Single-cell suspensions were obtained by passing the cells through a 70 μm BD Falcon strainer (Becton & Dickinson, Sunnyvale, CA). After filtration, the cell suspensions were seeded in a 100 mm dish containing α-MEM supplemented with 10% fetal bovine serum (FBS). The culture dishes were incubated at 37°C and the medium was changed every third day. DPSCs/progenitors prepared in this manner characteristically show a high expression of typical mesenchymal stem cell markers; CD29: 96.2%, CD44: 91.1%, CD105: 95.0%, CD90: 95.7% [20]. DPSCs/progenitors under passage 5 were used in this study.

To induce odontoblastic differentiation, DPSCs/progenitors were seeded at 10,000 cells/cm^2^ (55 ~ 60% confluence) and grown in a differentiation medium, consisting of α-MEM supplemented with 10% FBS, 50 μg/mL ascorbic acid, 10 mM β-glycerophosphate, and 10^−8^ M dexamethasone (Sigma-Aldrich, USA).

### Microarray analysis

Human DPSCs/progenitors were cultured in differentiation media for 1 or 7 days. One microgram of extracted RNA was sent to Seoulin bioscience (Gyeonggi-do, Korea) for microarray analysis. Microarray analysis was performed using the Affymetrix HGU133 GeneChip Array platform (Affymetrix, Santa Clara, CA). The results obtained were validated using a reverse transcriptase–polymerase chain reaction (RT-PCR).

### RNA isolation and RT-PCR amplification

The expression of genes related to odontoblast differentiation, such as ALP, OPN, BSP, DMP1 and DSPP, were analyzed by RT-PCR. Mouse tissues were prepared following approval by and guidance of the Institutional Ethics Committee of Kyungpook National University. The total RNA was extracted using the Tri reagent (Molecular Research Center, Inc., Cincinnati, OH), and the cDNA was synthesized from 1 μg of RNA using Superscript II (Invitrogen, Carlsbad, CA). The following thermal cycling parameters were used for PCR: 95°C for 2 min, followed by the appropriate number of cycles of 95°C for 45 s, the appropriate annealing temperature for 45 s, 72°C for 60 s, and a final extension at 72°C for 5 min. Human glyceraldehyde-3-phosphate dehydrogenase (GAPDH) was used as the internal control. Sequences of PCR primers were shown in Additional file 1: Table S1. All PCR products were subjected to 1% agarose gel electrophoresis and visualized by ethidium bromide staining. The gels were photographed, and the expression levels of the genes were quantified using the Wisdoc system (DAIHAN-Sci., Seoul, Korea).

### Proliferation assay

The growth rate of the DPSCs/progenitors electroporated with BBX variant 1 and 2 cDNAs or shRNA was evaluated at days 0, 3, 6, and 9. After electroporation, methyl-thiazol tetrazolium (MTT) assay (Sigma-Aldrich) was performed as described previously [32]. Total cell number was also counted using a hemocytometer.

### Immunocytochemistry

Human DPSCs/progenitors were cultured in growth media or differentiation media for 14 days. The cells were fixed with 4% paraformaldehyde and incubated in 0.25% TritonX-100 for permeabilization. The cells were incubated with rabbit anti-BBX antibody (1:100) (Abcam, Cambridge, MA) followed by Alexa Fluor 488-conjugated anti-rabbit secondary antibodies (Invitrogen). Nuclei were stained with 1 μg/ml DAPI. Images were taken with a confocal laser microscopy (Carl Zeiss, Germany).

### Alizarin red S staining

Alizarin red S staining was used to evaluate the mineralization of DPSCs/progenitors. At 25~30 days after odontoblast differentiation medium stimulation, the cells were fixed in 70% EtOH for 10 minutes. After washing with distilled water, the cells were stained with an Alizarin Red Solution (2%, pH 4.2) (Sigma-Aldrich, USA) for 10 min, followed by extensive washing with distilled water to remove the unbound stain.

### ALP staining

ALP staining was performed according to the manufacturer’s instructions. The medium of the culture dish was aspirated. After fixing with citrate solution, the cells were incubated with an alkaline phosphatase substrate, NTB/BCIP (Nitro Blue Tetrazolium/ 5-bromo-4-chloro-3′-indolyphosphate), for 20 min. The ALP positive cells were stained a purple color.

### Electroporation

The BBX variant 1 and 2 full length cDNA molecules were cloned from the cDNA of DPSCs/progenitors undergoing odontoblast differentiation. FLAG tagged-BBX variant 1 and 2 cDNAs were cloned into pMXs-IRES-Blasticidine vector (Cell Biolabs, San Diego, CA), and verified by DNA sequencing. The BBX shRNAs was purchased from Open Biosystems (Termo scientific, Waltham, MA). The sequences of BBX and control (N/C) shRNA was 5′-GACCTTATCTTCCTTATAT-3′ and 5′-GGAATCTCATTCGATGCATAC-3′, respectively. The wild-type BBX variant 1 and 2 cDNAs and BBX shRNA was electroporated into the DPSCs/progenitors using a Microporator (Invitrogen). Briefly, the cells (2.0 × 10^5^) were transfected with 1 μg of the BBX variant 1 or 2 cDNA using a 950 pulse voltage, 30 pulse width and 2 pulse. The viability of cells ranged between 60 and 65% following electroporation.

To examine the transfection efficiency, DPSCs/progenitors were transfected with FLAG tagged BBX variant 1 vector, and two days later, immunofluorescence staining with FLAG antibody was performed. The result showed that approximately 80% of DPSCs/progenitors were positive for FLAG (Additional file 4: Figure S3).

## Abbreviations

DPSC: Dental pulp stem cells; BBX: Bobby sox homolog; ALP: Alkaline phosphatase; DSPP: Dentin sialophosphoprotein; DMP1: Dentin matrix protein 1.

## Competing interests

The authors declare that they have no competing interests.

## Authors’ contributions

YAC and MYS: conception, design of experiment, collection and/or assembly of data, data analysis and interpretation. HIS: interpretation and discussion. EKP: conception and design, data analysis and interpretation, preparation of the article. All authors read and approved the final manuscript.

## Supplementary Material

Additional file 1: Table S1 Sequences of primers, product size and annealing temperature of RT-PCR. Click here for file

Additional file 2: Figure S1 The expression of flag tagged-BBX at day 14. Click here for file

Additional file 3: Figure S2 BBX expression was analyzed in BMSCs undergoing osteoblast differentiation. Click here for file

Additional file 4: Figure S3 Transfection efficiency was checked at day 2 after electroporation. Click here for file
