# Supplementary material for: Bobby Sox homology regulates odontoblast differentiation of human dental pulp stem cells/progenitors
Source: Cell Commun Signal. 2014 May 30;12:35. doi: 10.1186/1478-811X-12-35 (PMC4062286; doi:10.1186/1478-811X-12-35)
Supplement: Additional file 1: Table S1 — Sequences of primers, product size and annealing temperature of RT-PCR. [file 1478-811X-12-35-S1.docx]

Additional file 1: Table S1. Sequences of primers, product size and annealing temperature of RT-PCR.

| Gene | F/R | Sequence | Product size (bp) | Annealing temp |
| --- | --- | --- | --- | --- |
| BBX total | F | 5’-TTGCAAACGCCATCGCTCTCTT-3' | 586 | 57 |
|  | R | 5’-ACCACCCAACTGCGAAGTGTTT-3' |  |  |
| BBX v1/v2 | F | 5’-CTGATCATGAAGGGTGTTGGA-3' | 444 / 435 | 59 |
|  | R | 5’-CAGGCTCCATGGTGTTTCTG-3' |  |  |
| BBX v1/v2/v3 | F | 5’-CTGAGCCTGTAAAACGCTGRG-3' | 1531/1441  /522 | 57 |
|  | R | 5’-CAGGCTCCATGGTGTTTCTG-3' |  |  |
| SATB1 | F | 5’-TGATGGCTCAGCTGCTGAAC-3' | 631 | 58 |
|  | R | 5’-AACAGCTCGCACAACCATCC-3' |  |  |
| ZBTB38 | F | 5’-AGTGTTCGATGACGCAAGTG-3' | 331 | 53 |
|  | R | 5’-GGCATATGGCCGAGAAGTAA-3' |  |  |
| ZBTB20 | F | 5’-GGCTGAACCCACCCAACCCG-3' | 386 | 61 |
|  | R | 5’-GGGGCTGTGGCAGGCTGAAG-3' |  |  |
| ZFP90 | F | 5’-GAGGGAGCTTTCCCTAATGG-3' | 381 | 55 |
|  | R | 5’-TCCACTGTGTGTGACCTCGT-3' |  |  |
| PBX1 | F | 5’-AGCATCATCCACCGCAAGTT-3' | 358 | 55 |
|  | R | 5’-TTCCATGGGCTGACACATTGGT-3' |  |  |
| SSBP2 | F | 5’-CAGAACTATGGAGGTGCAAT-3' | 341 | 51 |
|  | R | 5’-TGTGACTCCATTCCTCCTAA-3' |  |  |
| BSP | F | 5’-AATGAAAACGAAGAAAGCGAAG-3' | 450 | 53 |
|  | R | 5’-ATCATAGCCATCGTAGCCTTGT-3' |  |  |
| OPN | F | 5’-AGCCAGGACTCCATTGACTCGAAC-3' | 416 | 58 |
|  | R | 5’-GTTTCAGCACTCTGGTCATCCAGC-3' |  |  |
| ALP | F | 5’-ACCATTCCCACGTCTTCACA-3' | 162 | 55 |
|  | R | 5’-AGACATTCTCTCGTTCACCG-3' |  |  |
| DMP1 | F | 5’-CAGGAGCACAGGAAAAGGAG-3' | 213 | 59 |
|  | R | 5’-CTGGTGGTATCTTGGGCACT-3' |  |  |
| DSPP | F | 5’-CAACCATAGAGAAAGCAAACGCG-3' | 120 | 56 |
|  | R | 5’-TTTCTGTTGCCACTGCTGGGAC-3' |  |  |
| GAPDH | F | 5’-CGACCACTTTGTCAAGCTCA-3' | 203 | 56 |
|  | R | 5’-AGGGGAGATTCAGTGTGGTG-3' |  |  |
