# Supplementary figures and images for: Bobby Sox homology regulates odontoblast differentiation of human dental pulp stem cells/progenitors
Source: Cell Commun Signal. 2014 May 30;12:35. doi: 10.1186/1478-811X-12-35 (PMC4062286; doi:10.1186/1478-811X-12-35)

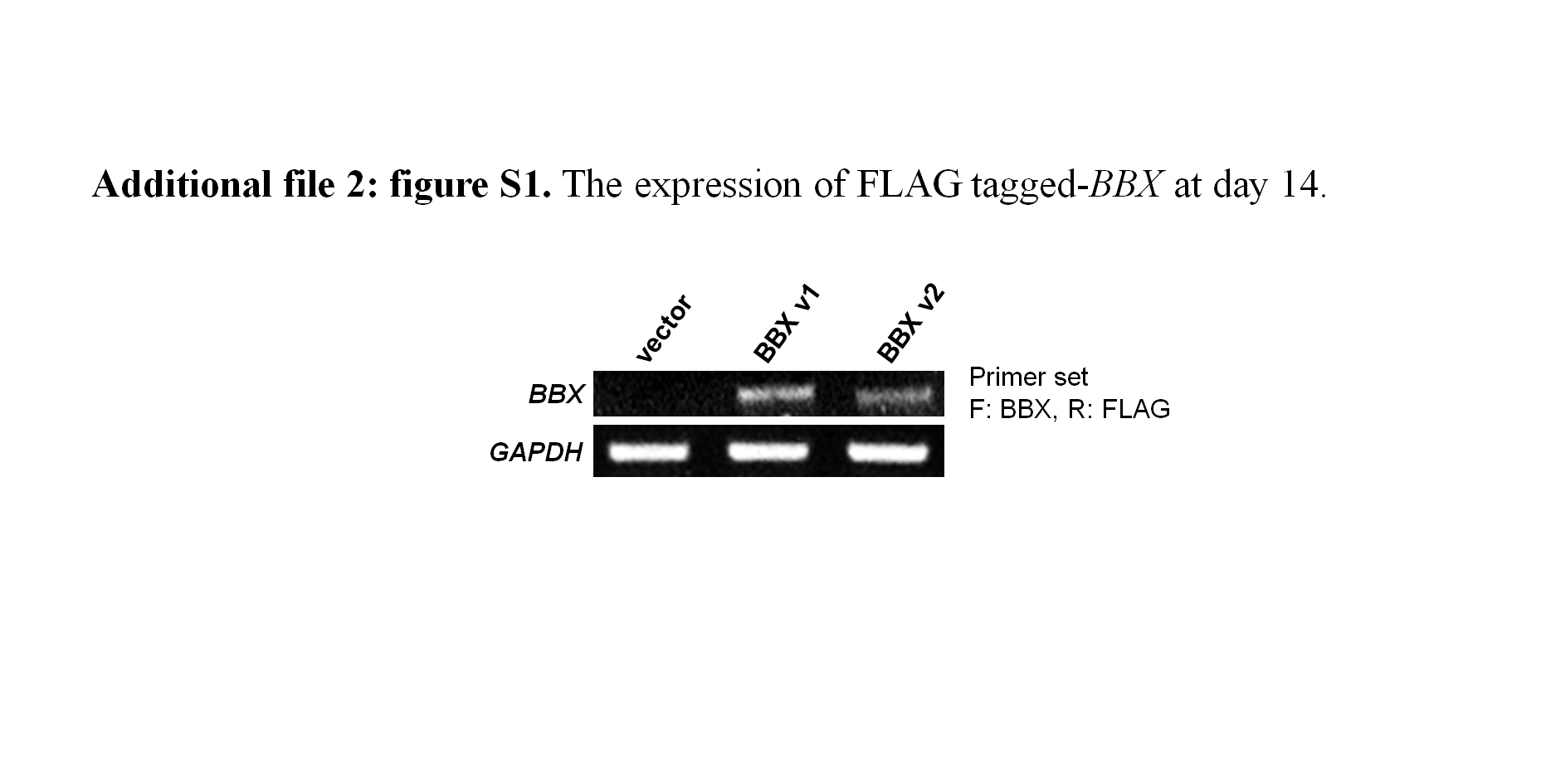

Supplement: Additional file 2: Figure S1 — The expression of flag tagged-BBX at day 14. [file 1478-811X-12-35-S2.jpeg]

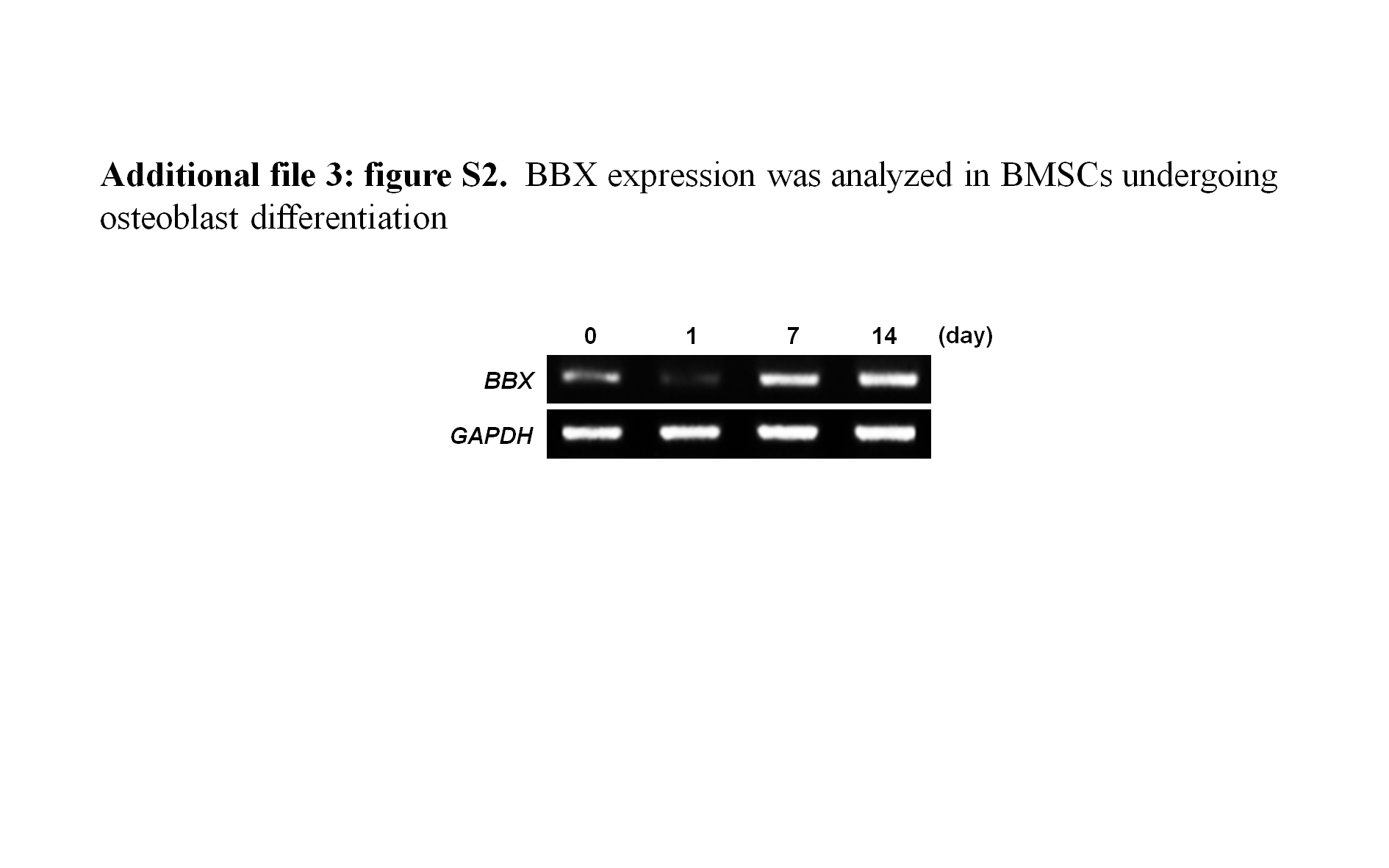

Supplement: Additional file 3: Figure S2 — BBX expression was analyzed in BMSCs undergoing osteoblast differentiation. [file 1478-811X-12-35-S3.jpeg]

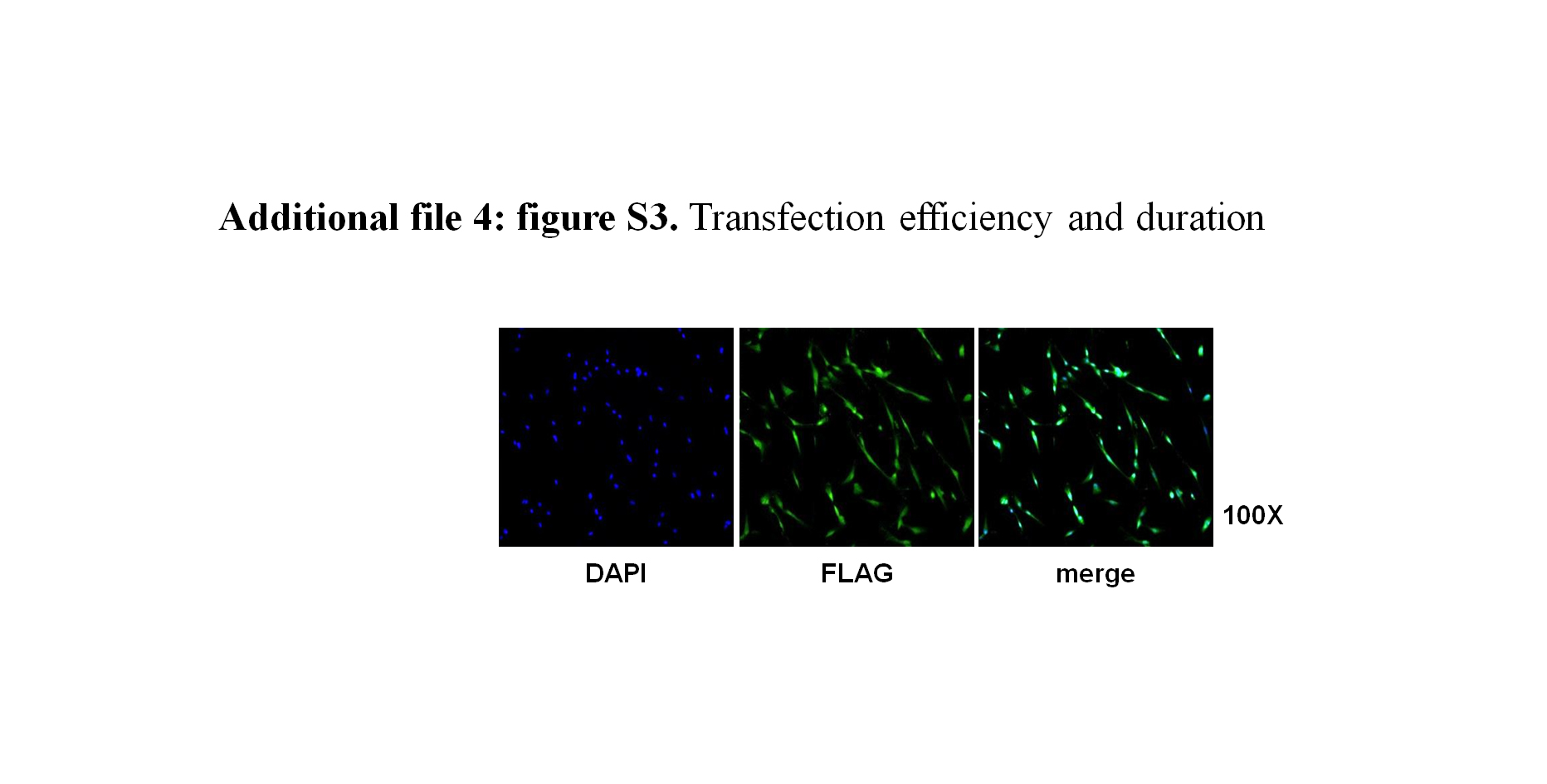

Supplement: Additional file 4: Figure S3 — Transfection efficiency was checked at day 2 after electroporation. [file 1478-811X-12-35-S4.jpeg]
